# Machine Learning Improves Upon Clinicians' Prediction of End Stage Kidney Disease

**DOI:** 10.3389/fmed.2022.837232

**Published:** 2022-03-16

**Authors:** Aaron Chuah, Giles Walters, Daniel Christiadi, Krishna Karpe, Alice Kennard, Richard Singer, Girish Talaulikar, Wenbo Ge, Hanna Suominen, T. Daniel Andrews, Simon Jiang

**Affiliations:** ^1^Department of Immunology and Infectious Disease, John Curtin School of Medical Research, Australian National University (ANU), Canberra, ACT, Australia; ^2^Department of Renal Medicine, The Canberra Hospital, Garran, ACT, Australia; ^3^School of Computing, Australian National University, ACT, Australia; ^4^Department of Computing, University of Turku, Turku, Finland; ^5^Centre for Personalised Immunology, Australian National University (ANU), Canberra, ACT, Australia

**Keywords:** machine learning (ML), prediction model, end stage kidney disease (ESKD), XGBoost (Extreme Gradient Boosting), chronic kidney disease

## Abstract

**Background and Objectives:**

Chronic kidney disease progression to ESKD is associated with a marked increase in mortality and morbidity. Its progression is highly variable and difficult to predict.

**Methods:**

This is an observational, retrospective, single-centre study. The cohort was patients attending hospital and nephrology clinic at The Canberra Hospital from September 1996 to March 2018. Demographic data, vital signs, kidney function test, proteinuria, and serum glucose were extracted. The model was trained on the featurised time series data with XGBoost. Its performance was compared against six nephrologists and the Kidney Failure Risk Equation (KFRE).

**Results:**

A total of 12,371 patients were included, with 2,388 were found to have an adequate density (three eGFR data points in the first 2 years) for subsequent analysis. Patients were divided into 80%/20% ratio for training and testing datasets.

ML model had superior performance than nephrologist in predicting ESKD within 2 years with 93.9% accuracy, 60% sensitivity, 97.7% specificity, 75% positive predictive value. The ML model was superior in all performance metrics to the KFRE 4- and 8-variable models.

eGFR and glucose were found to be highly contributing to the ESKD prediction performance.

**Conclusions:**

The computational predictions had higher accuracy, specificity and positive predictive value, which indicates the potential integration into clinical workflows for decision support.

## Introduction

Chronic kidney disease (CKD) is a major cause of morbidity and mortality globally, having a reported prevalence of 11–13% (1). The prevalence of CKD is rising, especially in developed nations where lifestyle related diseases are endemic (2). Whilst CKD may ultimately culminate in end stage kidney disease (ESKD), rate of progression is highly variable and difficult to predict. ESKD is associated with a marked increase in mortality and morbidity: it is a terminal condition without renal replacement therapy (RRT) in the form of haemodialysis, peritoneal dialysis, or kidney transplantation. As ESKD approaches, patients and clinicians are required to make difficult decisions (3). RRT requires the formation of permanent dialysis access and/or evaluation of suitability for transplantation. Preparation for RRT is associated with significant cost and risks of complications, such as post-operative infection and bleeding (4). Premature access formation exposes patients to these risks without benefit and, in some cases, result in RRT access not being used at all (5). However, the capacity of physicians to correctly predict patient outcomes is poor (6). Therefore, any method which will improve the ability to correctly identify patients who will require RRT is highly desirable.

Data-driven predictive modeling is a rapidly advancing field and has been employed in a range of clinical scenarios such as opioid overdose (7) and acute kidney injury (8). Recent advances have demonstrated the capacity of predictive modeling to robustly predict acute kidney injury in individuals with varying levels of kidney function (8). For CKD, multiple risk factors for initiation and progression to ESKD have been characterized (9). The most pronounced risk factors include male gender, proteinuria, excess weight, hypertension and diabetes (10, 11). Statistical modeling of these data has been shown to be predictive of an individual developing CKD (12, 13). Application of machine learning (ML) approaches in small clinical cohorts has been shown to be capable of predicting accurate estimated glomerular filtration rate (eGFR) values (14, 15). Analysis of over sixty thousand electronic medical records (EMRs) and prediction with a random forest regression method has shown eGFR to be predicted with a correlation coefficient of better than 0.95 (16). However, to date, ML has not been used to predict the clinical outcomes of ESKD or death in CKD. It has also not been compared pragmatically to the predictions of expert clinicians.

In this work, we describe the training of a ML model that was capable of predicting which CKD patients will progress to ESKD within 2 years. We compare the predictive power of this model against the prospective predictions of six nephrologists in a cohort of fifty CKD patients. The predictions for ESKD occurring within a 2-year period were better than the most experienced clinician. The work here shows that predictive models built with machine learning can be accurate and have a potential role in providing decision support in a clinical environment.

## Methods

### Data Cleaning

The raw clinical time-series data included much manually entered information, and included erroneous extreme outliers in some variables, particularly, height, weight, standing systolic pressure, standing heart rate and protein-creatinine. These were outliers detected and removed using chi-squared outlier tests with an inclusion threshold of 99.999999%, excluding points with a *p*-value of lesser than 10^−8^ for distribution membership. A total of 538 outlying points were flagged, out of 869,901 data points (0.062%).

### Data Filtering

Out of 12,371 patients extracted 7,565 patients recorded an eGFR value. We filtered the primary data to remove patients with fewer than three pre-ESKD eGFR measurements. This filtering step retained 4,477 patients. Primary measures of creatinine level were excluded from subsequent study due to the output predictor, eGFR, being derived from it.

### Machine Learning Model

The Training Span (*TS)* and the Test Timepoint (*TT*) values were chosen as a trade-off between accuracy and the clinical usefulness of the size of the prediction window. Here, *TS* = 2 and *TT* = 8 years provided the most clinically relevant predictor, with a large amount of useful training data and relatively high accuracy. With this selection of *TS*, we removed patients who had reached ESKD within 2 years of commencement of their data collection. This reduced our sample size to 2,388 patients.

### Data Partitioning

To ensure that the model was trained on distinct data to the test data, individual patients were fist randomly partitioned into two groups. 80% (*N* = 1,910) of the patients formed the training set and the remaining 20% (*N* = 478) was used for testing. To ensure that a similar proportion of ESKD patients were randomly included in each set, a two-sample test for equality of proportions with continuity correction was performed, which confirmed that the proportions were not significantly different (χ^2^ = 0.000538, *p* = 0.9812).

### Time-Series Featurisation

Data from 19 different time-series from each of the 1,910 training and 478 test patients was featurised with the tsfresh v0.12.0 python library (17), using the internal *Comprehensive* calculator setting to produce 764 features per individual timeseries (including power spectral density, Fourier, cosine and wavelet transforms). Four case studies with varying degrees of *Comprehensive* featurisation was performed: zero (all *Minimal*), 2 (only eGFR and glucose), 6 (adding standing heart rate, systolic and diastolic blood pressure and heart rate), and 19 (all *Comprehensive*).

### Model Training and Hyperparameter Tuning

A model was trained on the featurised time series data for the 1,910 training set patients with XGBoost (18) (version 0.90.0.2), as implemented in R 3.6.1. This method is particularly suited to efficiently handle sparse data. Since the prediction accuracy of XGBoost can vary greatly according to various hyperparameters, we performed 10-fold cross-validation (CV) and hyperparameter grid search across five frequently-cited (19) tunable XGBoost parameters (*nrounds, eta, max_depth, subsample, colsample_bytree*). In order to properly handle the over 8-fold class imbalance between ESKD cases (*N* = 263) and non-ESKD cases (*N* = 2,125), the metric for tuning and evaluating performance of the predictive models was changed from accuracy to Area Under the Precision-Recall Curve, AUCPR (20). Upon subsequent prototyping, the Matthews Correlation Coefficient (MCC) metric, reported to be optimal for imbalanced binary classification (21), indeed performed better than AUCPR on our dataset and was solely employed in all subsequent analyses. The highest 10-fold CV mean MCC of 0.4327 corresponded with *nrounds* = 200, *eta* = 0.01, *max_depth* = 4, *subsample* = 1, *colsample_bytree* = 0.8, and this hyperparameter set was used to build the final optimal model employing the entire training dataset. The fully-featurised (all-Comprehensive) run had a 10-fold CV mean MCC of 0.4303 with the corresponding best hyperparameter set: *nrounds* = 500, *eta* = 0.04, *max_depth* = 4, *subsample* = 1, *colsample_bytree* = 0.6. SHapley Additive exPlanation (SHAP) values (22) were calculated and graphed using the SHAPforxgboost 0.0.2 R library (23). Our software and trained models are available for download from (https://github.com/catlyst/trendal).

## Results

### Dataset

We obtained pathology and clinical records for patients attending hospital and outpatient clinics at The Canberra Hospital Department of Renal Medicine, Australian Capital Territory, Australia. This included 12,371 patients spanning 21.5 years from September 1996 to March 2018. In addition to birth year, sex and date of death, this dataset consists of 17 time-stamped clinical measures including creatinine and derived eGFR (168,500 points each), glucose (145,961 points), sitting and standing blood pressure (42,818 points), heart rate (28,741 points), weight (47,981 points), height (35,421 points) and derived Body Mass Index (BMI), HbA1c (15,349 points), urine protein-creatinine ratio (14,777 points) and 24-h proteinuria (883 points). All data was observed in a normal distribution with the exception of creatinine, eGFR, glucose, HbA1c, proteinuria and urine protein/creatinine ratio (Supplementary Figure 1). In addition to the clinical measures, we derived two features, sitting and standing pulse pressure. With the addition of these derived measures, a total of 19 time-based measurements were obtained. We applied a threshold of three eGFR data points obtained in the first 2 years for each individual as a required minimum data density for subsequent analysis. From 7,565 patients, 2,388 were found to have adequate density for ML prediction (31.6%) (Supplementary Figure 2).

Out of 2,388 patients, 1,910 were used to train the model and 478 were used as a holdout test dataset. Patients baseline characteristics are shown in Table 1. There was no difference in the age at presentation, gender proportion, diagnosis, and CKD stage. The majority of patients were in CKD stage 2 and 3.

**Table 1:** Baseline characteristics.

	**Train,** ***N* = 1,910**	**Test,** ***N* = 478**	***P*-value**
Age at presentation (median, IQR)	62 (48–72)	64 (51–73)	0.13
Sex Male	1,101 (58%)	274 (57%)	0.9
Diagnosis			0.8
DKD	412 (22%)	112 (23%)	
HTN	357 (19%)	83 (17%)	
GN and Vasculitis	272 (14%)	65 (14%)	
Genetic	50 (2.6%)	10 (2.1%)	
Other	819 (43%)	208 (44%)	
CKD Stage			0.14
Stage 1	137 (7.2%)	20 (4.2%)	
Stage 2	571 (30%)	153 (32%)	
Stage 3a	379 (20%)	102 (21%)	
Stage 3b	489 (26%)	114 (24%)	
Stage 4	334 (17%)	89 (19%)	

*P-values reported are for chi-squared tests of homogeneity between the train and test sets. Since these p-values are insignificant (above 0.05), the train and test sets are deemed homogeneous. CKD, Chronic Kidney Disease; DKD, Diabetic Kidney Disease; GN, Glomerulonephritis*.

### Machine Learning Model Identifies EGFR and Cardiovascular Risk Factors as Key Predictors of ESKD

We aimed to develop a model capable of predicting whether a patient would reach ESKD and to estimate the timeframe in which this would occur. We first aggregated individual patients' clinical measurements into a single*, initial* predictive model, including potentially interacting and confounding variables (see *Methods*). Throughout this analysis, we defined ESKD as the composite endpoint of an eGFR below a threshold of 10 mL/min/1.73 m^2^ (24) or the commencement of RRT, whichever came first. Using the *initial* model, we then incorporated data for each patient with an observation period, or *training span* (*TS*), of 2 years to predict if the patient would reach ESKD by a *test timepoint* (*TT*) of 8 years. The *initial* model incorporated all 19 time series measures present in the primary data and this was augmented with full featurisation derived from these timeseries (see *Methods*). When predicting if ESKD would occur by 8 years follow-up, the *initial* model had a prediction accuracy for ESKD of 84.5% with a sensitivity and specificity of 55.8 and 88.0%, respectively.

To understand the relative contribution of each feature in the *initial* model, SHapley Additive exPlanation (SHAP) values (25) were calculated, representing the weight of a particular feature in the model's ESKD prediction outcome. A SHAP value >0 suggests the feature value is a risk for ESKD, and <0 suggests the feature value is protective against the risk of ESKD.

**Figure 1:**
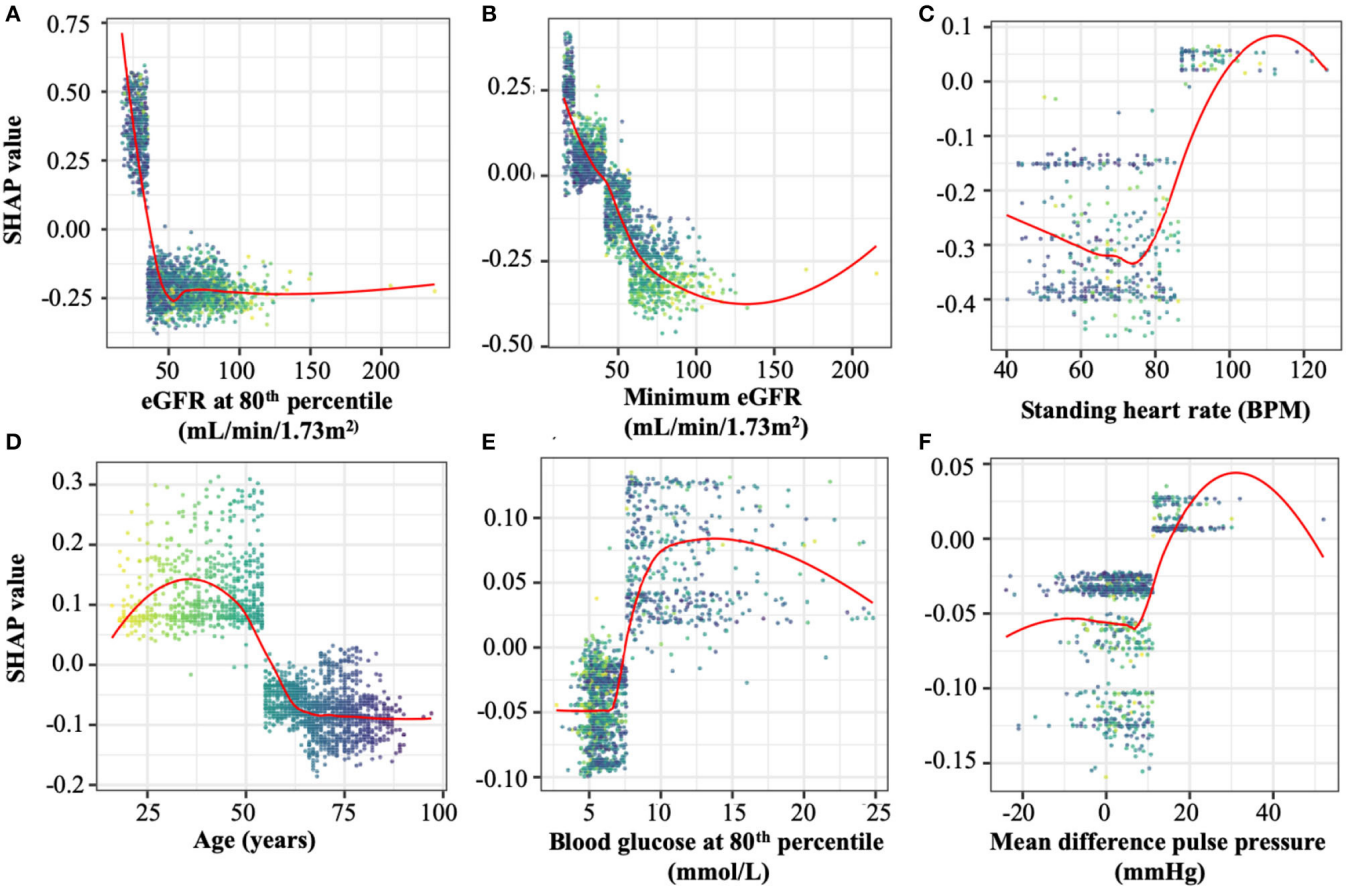
Representative SHAP dependency plots taken from the top 25 features in the optimal model. SHAP values represent the predictive value of a feature in models in which they are integrated. Positive SHAP valu es imply a contribution to ESKD risk, while negative values are protective against ESKD. Selected panels are **(A)** estimated glomerular filtration rate (eGFR) at the 80th percentile, the most predictive of ESKD in both models, **(B)** minimum eGFR, the second-most predictive value in both models, **(C)** Standing heart rate, **(D)** patient age at study initiation, **(E)** blood glucose levels at the 80th percentile, and **(F)** individual points represent the SHAP and feature values of an individual evaluated by the optimal model. The points in all plots are colored spectrally by initial age to differentiate between younger patients (yellow) and older patients (dark blue). The top 25 SHAP dependency plots are shown in full in Supplementary Figure 3.

For the prediction of ESKD, as expected eGFR-based features (the minimum value and values at the 80, 20, and 10th percentiles) had the highest contributions, comprising six of the top 10 features (Table 2). Other quantities present in the top 10 predictive features included the patient's minimum standing heart rate, their age at first consultation and several derived or transformed values related to blood glucose levels and eGFR (Figure 1; Supplementary Figure 3). Further, blood glucose was a major contributor to the prediction of ESKD (6 of top 25 features) when >7 mmol/L (Figure 1; Table 2). Other identified features contributing to risk include known clinical risk factors such as blood pressure features including minimum standing diastolic blood pressure where a value of >75 mmHg increases risk. The model also identified other features such as minimum heart rate (increased risk at more than ~85 bpm), age at initial attendance (increased risk, <55 yrs) and mean pulse pressure difference between sitting and standing (increased risk, >10) which are not known as clinical risk factors for ESKD but appear to have cutoff values associated with ESKD risk. We hypothesized that increased cardiovascular disease explained the association of increased HR and pulse pressure difference, and that the competing interest of death at higher age groups made youth a risk factor for progression to ESKD.

**Table 2:** The 25 most predictive features of the initial and optimized models.

**Data type**	**Featurised value**	**Rank** **(*initial* model)**	**SHAP value** **(*initial* model)**	**Rank** **(*optimized* model)**	**SHAP value** **(*optimized* model)**
eGFR	80th percentile	1	0.25254	1	0.24691
eGFR	Minimum	2	0.15594	2	0.14371
eGFR	20th percentile	3	0.13539	3	0.13772
eGFR	10th percentile	4	0.11008	4	0.11848
Standing heart rate	Minimum	5	0.09653	25	0.01417
Age	Initial	6	0.09118	5	0.08176
eGFR	CWT coefficients (2,5,10,20; 2,2)	7	0.05538	10	0.03539
Glucose	80th percentile	8	0.05421	8	0.05527
eGFR	70th percentile	9	0.04888	6	0.06204
eGFR	60th percentile	10	0.03283	14	0.02945
Standing-sitting pulse pressure difference	Mean	11	0.0327	–	–
eGFR	Mean change in 40 and 80th percentile	12	0.03149	11	0.03376
Glucose	CWT coefficients (2,5,10,20; 2,20)	13	0.02902	18	0.02302
Glucose	10th percentile	14	0.02861	–	–
Standing diastolic blood pressure	Minimum	15	0.02707	–	–
Glucose	Unnormalized CID Complexity Estimate	16	0.026	24	0.01522
Sitting heart rate	Minimum	17	0.02561	9	0.03748
eGFR	Mean central second derivative	18	0.02501	12	0.03189
eGFR	40th percentile	19	0.0224	–	–
eGFR	Mean change	20	0.02229	23	0.01572
Glucose	Absolute energy	21	0.02136	16	0.02408
eGFR	Linear trend rightmost-value	22	0.01957	15	0.02541
Sitting heart rate	Maximum	23	0.01931	–	–
Sitting systolic blood pressure	Minimum	24	0.01916	–	–
Glucose	Median	25	0.01889	21	0.01802
Sitting heart rate	Linear trend rightmost-value	–	–	7	0.06066
Sitting heart rate	Welch density (coeff = 2)	–	–	13	0.02951
Sitting systolic blood pressure	C3 non-linearity in timeseries (lag = 1)	–	–	17	0.02353
Glucose	10th percentile	–	–	19	0.023
Standing-sitting pulse pressure difference	CWT coefficients (2,5,10,20; 0,2)	–	–	20	0.02133
Glucose	Maximum	–	–	22	0.01694

Given the contribution of eGFR and glucose we modified our ML algorithm to emphasize these variables. We hypothesized that the prominence of eGFR and glucose suggested these variables contributed to the majority of the prediction. Further, we noted that the greatest data density existed for eGFR. We trained a further, *optimized* model utilizing all 19 time-series measures but only comprehensively-featurised two timeseries values: that of eGFR and glucose, which were featurised each into 764 numerical features. We then only minimally-featurised the remaining 17 timeseries values (eight numerical features each). In predicting ESKD at 8 years on 2 years of patient data, this *optimized* model improved prediction accuracy from 84.5 to 86.2%. Sensitivity improved from 55.8 to 65.4% and specificity from 88 to 88.7%. The most predictive quantities identified from this *optimized* model are only slightly different (Supplementary Table 1) from the initial model. Therefore, we concluded that unbiased predictions of ESKD by our ML algorithm is based on classic ESKD risk factors and has a strong predictive capability.

To contrast optimized model performance by disease severity, we grouped patients in the test set by their eGFR at presentation (either below, or at least, 30 ml/min/1.73 m^2^) (Supplementary Table 2). Out of 478 patients in the test set, 90 (18.8%) had presenting eGFR below 30 ml/min/1.73 m^2^, of which 26 patients (28.9%) developed ESKD within 8 years. In contrast, of the 388 patients with presenting eGFR of at least 30 ml/min/1.73 m^2^, only 6.7% developed ESKD.

The model's accuracy in predicting ESKD in the former group was 67.8% (84.6% sensitivity, 60.9% specificity). In contrast, the accuracy of the model in the latter group was 90.4% (46.2% sensitivity, 93.6% specificity). These numbers highlight the sensitivity-specificity trade-off, which our single optimized model attempts to finely balance (overall accuracy 86.2% at 65.4% sensitivity and 88.7% specificity), but also suggests room for stratified ensemble models for future work.

### Comparison of ML Algorithm Against Clinician Prediction

**Figure 2:**
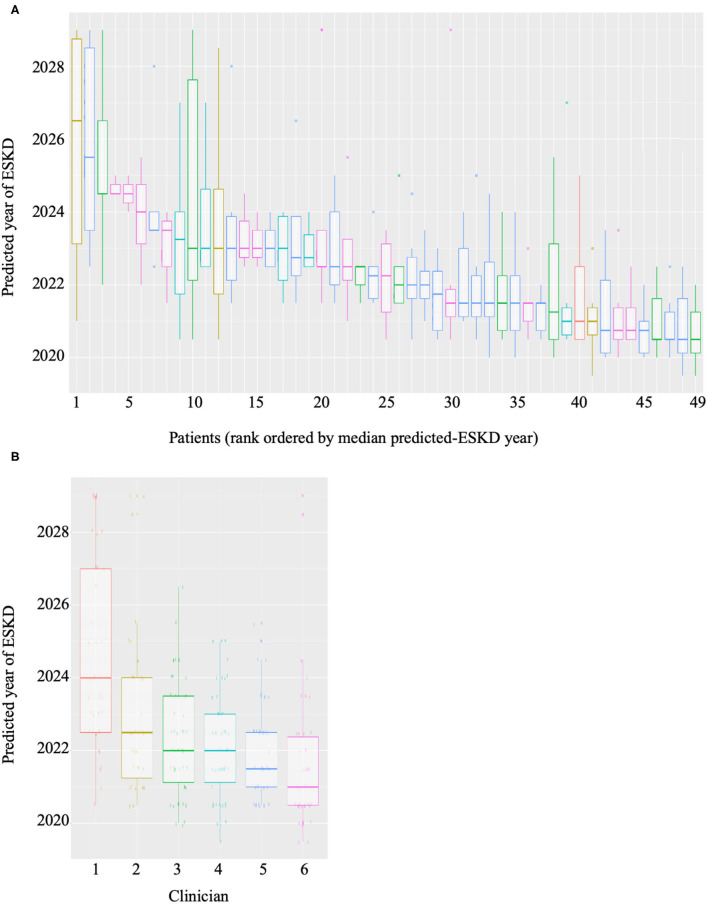
Boxplots of **(A)** predictions of year of ESKD for each patient by all six clinicians and **(B)** predictions of ESKD across the patient cohort by each clinician.

Forty-nine patients with CKD at risk of ESKD were selected as a test cohort. We considered the risk of ESKD at 2 years to be of greater clinical significance for RRT planning. Therefore, 2 years of clinical data was utilized by ML algorithm to provide a probability of reaching ESKD within the next 2 years. Six consultant nephrologists were given baseline demographics and diagnosis, medical comorbidities, proteinuria, blood pressure, and eGFR measurements over 2 years for the same cohort. The nephrologists predicted prospectively for each individual, within a 10-year window, the number of years (to the nearest half-year) it would likely take for the patient to reach ESKD. The ML algorithm and clinician predictions were compared to the outcomes in the 49 patients at the 2-year mark (the time of writing). The variability in the clinician predicted ESKD date for each patient was high (Figure 2A). As expected each clinician appeared to have a systematic bias, predicting longer or shorter times until ESKD was reached for the same cohort (Figure 2B). Variation was also high in the number of predictions made, with some clinicians predicting that all patients will reach ESKD, while others did not. The pairwise-correlations in the predicted dates of each clinician with correlation-coefficients ranging from 0.257 to 0.757 (Supplementary Figure 4). Clinician performance compared to the predictive model was poor (Table 3). The ML algorithm demonstrated a 2-year predictive accuracy of 93.9%, with sensitivity of 60%, specificity of 97.7% and precision (or positive predictive value) of 75%. This was superior to all clinicians, with the best performance being Clinician 4, who had an accuracy of 79.6% (sensitivity 80%, specificity 79.5%, precision 30.8%). All other clinicians performed significantly worse, primarily due to higher false positive counts (Table 3). Cumulatively, this data suggests that trained predictive ML modeling may assist nephrologists in making predictions of ESKD.

**Table 3:** Comparative performance of 2-year ESKD predictions by optimized model and expert clinicians.

**Metric**	**Prediction with optimized model**	**Clinician 1**	**Clinician 2**	**Clinician 3**	**Clinician 4**	**Clinician 5**	**Clinician 6**
Predicted ESKD (actual = 5/49 patients)	4	2	9	13	13	14	22
True positives	3	0	2	2	4	4	4
True negatives	43	42	37	33	35	34	26
False positives	1	2	7	11	9	10	18
False negatives	2	5	3	3	1	1	1
Accuracy	0.939	0.857	0.796	0.714	0.796	0.776	0.612
Sensitivity	0.600	0.000	0.400	0.400	0.800	0.800	0.800
Specificity	0.977	0.955	0.841	0.750	0.795	0.773	0.591
Precision (PPV)	0.750	0.000	0.222	0.154	0.308	0.286	0.182
F1 score	0.667	0.000	0.286	0.222	0.444	0.421	0.296
MCC	0.638	0.000	0.188	0.103	0.408	0.384	0.238

*ESKD, End Stage Kidney Disease; MCC, Matthews Correlation Coefficient; PPV, Positive Predictive Value*.

### Comparison of ML Algorithm Against Kidney Failure Risk Equations

The Kidney Failure Risk Equations (KFRE) (26) have been extensively used and validated to predict ESKD within 2 and 5 years. We used the updated 4- and 8- variable KFRE (13) to predict ESKD within 2 years for the test cohort of 49 patients, however as these equations rely on serum albumin tests, 3 patients had to be excluded (Table 4). The 4-variable KFRE (91.3% accuracy, 25% sensitivity, 97.6% specificity, 50% precision) performed better than the 8-variable KFRE (89.1% accuracy, 0% sensitivity, 97.6% specificity, 0% precision), which was consistent with validation studies (27), but both were inferior to our model.

**Table 4:** Comparative performance of 2-year ESKD predictions by optimized model and 4- and 8- variable KFRE.

**Metric**	**Prediction with optimized model**	**4-variable KFRE**	**8-variable KFRE**
Predicted end-stage (actual = 5/49 patients)	4	2	1
True positives	3	1	0
True negatives	43	41	41
False positives	1	1	1
False negatives	2	3	4
Accuracy	0.939	0.913	0.891
Sensitivity	0.600	0.250	0.000
Specificity	0.977	0.976	0.976
Precision (PPV)	0.750	0.500	0.000
F1 score	0.667	0.613	0.488
MCC	0.638	0.271	0.000

*ESKD, End Stage Kidney Disease; KFRE, Kidney Failure Risk Equation; MCC, Matthews Correlation Coefficient; PPV, Positive Predictive Value*.

### Other Metrics of Classification Performance

Like many other clinical predictive settings, one of the main challenges in building a reliable model was its class imbalance, e.g., in the clinician test cohort, only 5 out of the 49 patients actually reached ESKD, and even a null-predictor that predicts that none would reach ESKD would achieve a misleading accuracy of 89%. Commonly, F1 score (the harmonic mean of precision and recall) has been used to provide a fairer measure of predictor performance, but recently the Matthews correlation coefficient has been shown (28) to be better than both F1 and accuracy as a binary classification metric.

Revisiting our results (Tables 3, 4) shows that our model performs better at both F1 (0.667) and MCC (0.638) than the best of 6 clinicians (*F*1 = 0.444, MCC = 0.408) and the better of the KFRE predictors (*F*1 = 0.333, MCC = 0.271).

## Discussion

The prediction of hard outcomes of CKD such as death and ESKD is often inaccurate, especially progressing toward RRT (29, 30). Here we developed a predictive machine learning model that in our *optimized* model, comprising values from 19 dynamic measures including two fully-featurised time-series (eGFR and blood glucose), is able to predict the incidence of ESKD within an 8-year timeframe with an accuracy of 86.2%.

In a trial cohort of forty-nine patients assessed by six clinicians, the model was retrained to predict ESKD within a 2-year timeframe. The model proved to be more accurate and precise than all clinicians, however sensitivity was lower than some clinicians. Our model was trained from prospectively collected clinical data, and we showed that the 25 most predictive measures overlap with existing recognized clinical risk factors and recapitulate accepted clinical thresholds. For example, the inflection point for eGFR values is ~60 mL/min/1.73 m^2^ (stage 3A CKD) below which SHAP values increase sharply (31) and the inflection point of blood glucose (7 mmol/L) associated with risk of CKD progression is also accepted diabetic glucose treatment targets (32). Importantly, some measurements of accepted risk factor values (serum potassium, bicarbonate and uric acid) were not necessary or not included in our trained predictive models. This may indicate they closely replicate other information and trends already included in the models, or that fluctuations in these values are only weakly correlated with progression to ESKD.

The superior performance of the machine learning model compared to assessment by six experienced renal physicians leads supports the recognized variability of clinician performance. On the short-term outcome of the occurrence of ESKD within 2 years, clinician performance ranged from an accuracy of 47 to 81%.

Risk factors for ESKD have been extensively investigated in clinical studies and elevated blood pressure, proteinuria and kidney function are closely linked to ESKD (33). The description of risk factors, however, does not translate into an accurate assessment of future risk for an individual patient by clinicians, especially when accounting for the synergistic effects of these risk factors. Increasingly vast amounts of data accrued through medical records are difficult for clinicians to absorb, integrate and analyse into meaningful predictions.

We have purposefully restricted our analysis to factors known to be associated with an increased risk of ESKD to demonstrate the feasibility of using ML in a clinical setting to improve upon clinical prediction of hard endpoints. ML has the advantage of the ability to examine the features of the data that are most influential in the risk prediction. In our current model eGFR and glucose appeared to the be most influential. It is encouraging that specific parameters of risk factors appear to be linked to ESKD risk, such as diastolic blood pressure >75 mmHg and blood glucose over 7 mmol/L. This would suggest that the ML approach is able to detect appropriate clinical parameters.

The ML approach is also able to identify features that are not conventional risk factors for ESKD. In our current analysis, a sitting minimum heart rate below 75 bpm and an overall minimum heart rate below 85 bpm appears to be potentially protective, likely reflecting superior underlying cardiovascular health. This suggests ML may identify potential new clinical indicators of ESKD risk. Currently we have excluded other potential risk factors such as serum potassium, bicarbonate and uric acid, each of which have data suggesting they may be associated with risk of CKD progression.

Therefore, we conclude that unbiased ML modeling is capable of integrating large amounts of data points, establishing a predictive model that agnostically identifies classic cardiovascular ESKD risk factors to create robust predictions of ESKD. This forms the basis for improving prediction of ESKD to assist with RRT planning and prognostication, as well-potential identification of novel risk factors for ESKD.

## Data Availability Statement

The raw data supporting the conclusions of this article will be made available by the authors, without undue reservation.

## Ethics Statement

The studies involving human participants were reviewed and approved by ACT Health Research Ethics and Governance Office. Written informed consent for participation was not required for this study in accordance with the national legislation and the institutional requirements.

## Author Contributions

AC: developed the methods, analyzed the data, manuscript preparation, and review. GW: data extraction, advised on methods, and manuscript review. DC: manuscript preparation and review. KK, AK, RS, and GT: data generation/analysis and manuscript review. WG and HS: advised on methods and manuscript review. TA: supervision, manuscript preparation, and manuscript review. SJ: conceptualized the study, supervision, manuscript preparation, and manuscript review. All authors contributed to the article and approved the submitted version.

## Funding

SJ was supported by NHMRC project grant and Jacquot Research Establishment Grants. WG was supported by Australian Government Research Training Program Domestic Scholarship.

This research had been delivered in partnership with Our Health in Our Hands (OHIOH), a strategic initiative of the Australian National University, which aims to transform healthcare by developing new personalised health technologies and solutions in collaboration with patients, clinicians, and health care providers.

## Conflict of Interest

The authors declare that the research was conducted in the absence of any commercial or financial relationships that could be construed as a potential conflict of interest.

## Publisher's Note

All claims expressed in this article are solely those of the authors and do not necessarily represent those of their affiliated organizations, or those of the publisher, the editors and the reviewers. Any product that may be evaluated in this article, or claim that may be made by its manufacturer, is not guaranteed or endorsed by the publisher.
