# Supplementary material for: Machine Learning Improves Upon Clinicians' Prediction of End Stage Kidney Disease
Source: Front Med (Lausanne). 2022 Mar 16;9:837232. doi: 10.3389/fmed.2022.837232 (PMC8965763; doi:10.3389/fmed.2022.837232)
Supplement: Supplementary file 1 [file Data_Sheet_1.pdf]

Supplementary Figures

Supplementary Figure S1- Histograms of feature observations after outlier removal. The majority (9) of the features appear to be normally distributed. The non-normally distributed values are the laboratory-based measures which are positively skewed, due to their natural zero-minimum and open-ended maximum.

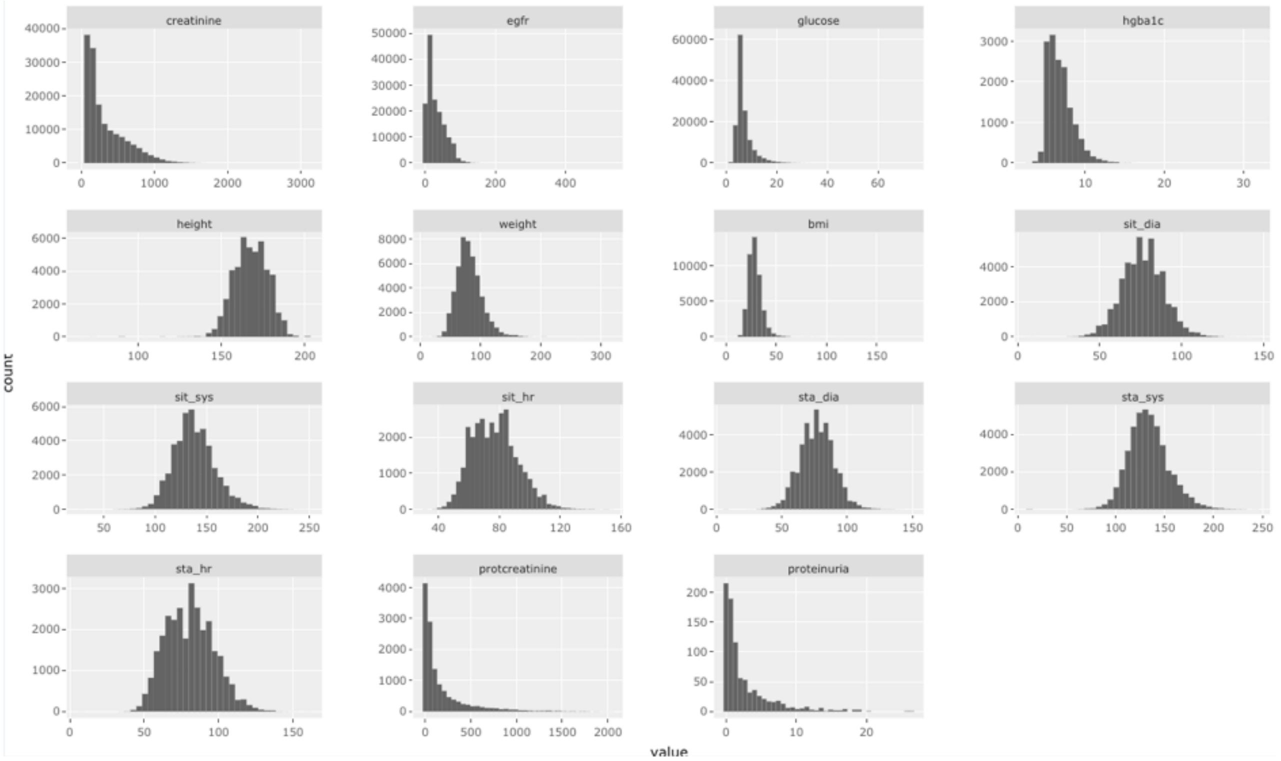

Supplementary Figure S2- Relative data densities of 15 features from the Canberra Hospital Renal database.

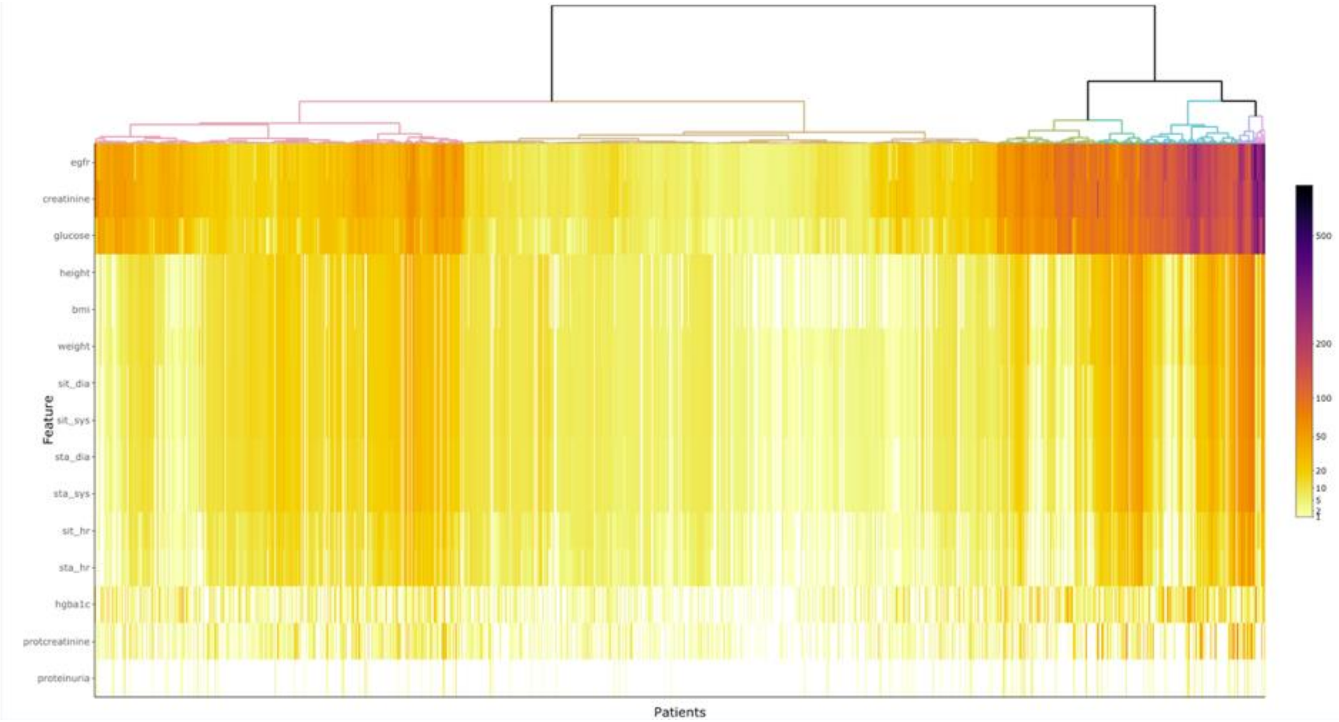

Supplementary Figure S3 - SHAP dependency plots of the top 25 features in the optimal model. SHAP values represent the importance of a feature, and the value of this feature, in predictive models in which they are integrated. These plots include the top 25 most-predictive features, ranked by SHAP values. Positive SHAP values imply a contribution to ESKD risk, while negative values are protective against ESKD. Individual points represent the SHAP and feature values of an individual evaluated by the optimal model. The points are coloured spectrally by initial age to differentiate between younger patients (yellow) and older patients (dark blue).

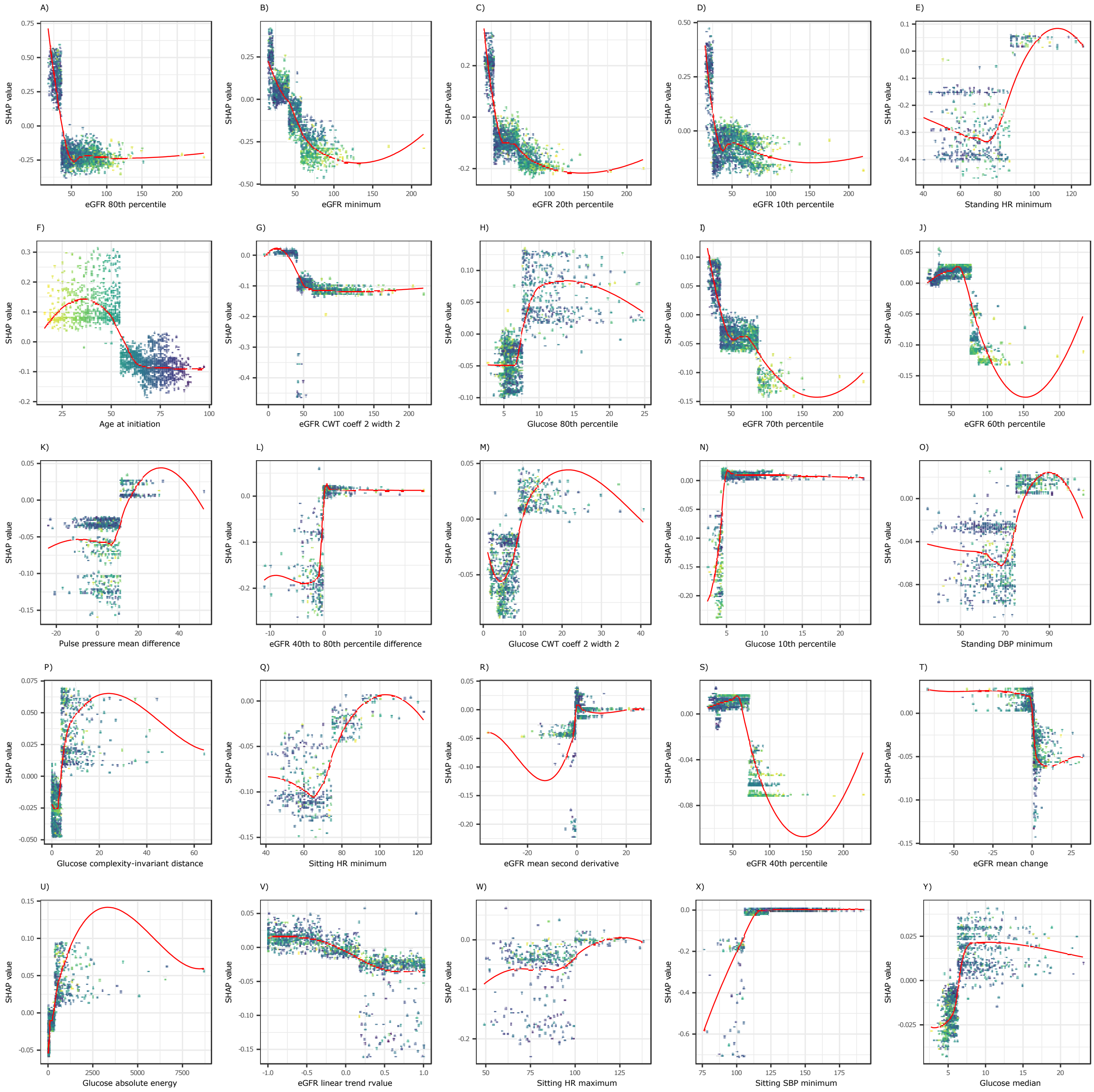

Supplementary Figure S4 - Pairwise correlation of clinician-predicted ESKD dates. When converting "2nd half of 2014" to a numeric year value, instead of picking the midpoint of the range (2014.75), we chose the most extant point of that half-year (2014.999+, i.e. 2015.0)

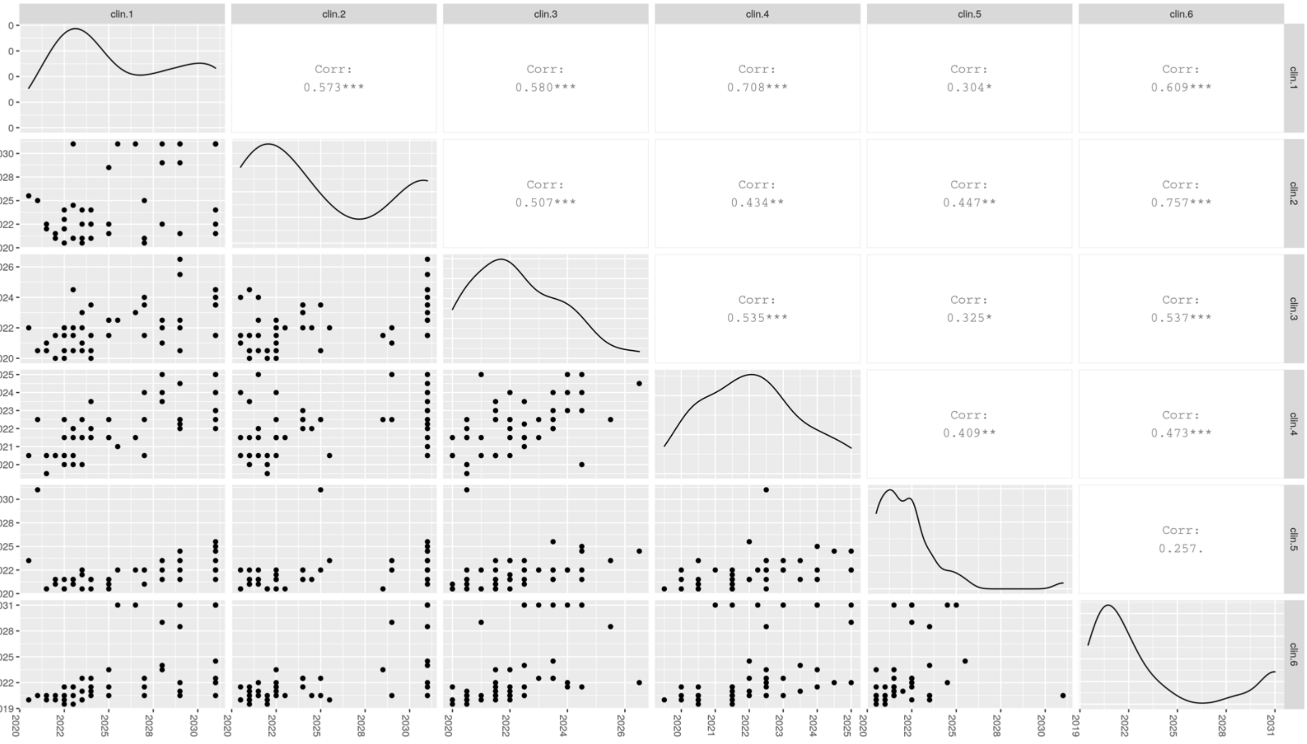

Supplementary Tables

Supplementary Table S1

Comparison of ML and clinician predictions of ESKD. Actual years to ESKD are highlighted in green, correct predictions are highlighted in yellow, and model prediction errors are in blue (false positive) and red (false negatives).

Age of Patient	Model prediction (probability of ESKD within 2 years)	Actual ESKD (years)	Clinician prediction (years until ESKD)					
			Clinician 1	Clinician 2	Clinician 3	Clinician 4	Clinician 5	Clinician 6
54	0.627	0.37	2.5	3	1.5	0.5	1.5	1
59	0.606	0.53	3.5	3	1.5	1	1.5	1.5
84	0.573		2.5	3.5	2	1.5	1.5	1.5
67	0.542	0.82	8	2	2.5	1.5	2	1.5
72	0.496	0.32	4	2	5.5	1	2.5	2.5
84	0.465		4.5	1.5	2	2.5	1.5	1
78	0.464		3.5	5	3	3.5	2.5	2.5
54	0.461		5	3.5	1.5	2.5	2.5	1.5
27	0.394		2	6	1.5	3.5	12	1.5
31	0.381		9	10	2	6	3.5	10
75	0.355		3.5	1.5	2.5	1.5	1.5	1
57	0.328		6	3.5	3.5	2.5	2.5	2.5
39	0.323		6	9.5	2.5	3.5	1.5	4.5
72	0.316		5	3.5	1	2.5	1.5	2.5
78	0.283		3.5	3	1	1.5	1.5	0.5
76	0.238		5	2	2.5	4.5	2.5	2
78	0.234		3	2.5	2.5	1.5	2.5	1.5
76	0.230		3	2	1	1.5	2	1
80	0.227		12	12	5.5	4	3.5	12
68	0.226		5	5	4.5	3	2.5	3.5
70	0.216		4.5	3.5	3	2.5	3	2
71	0.189	0.98	3.5	4	3	2.5	1.5	1.5
77	0.185		4	5.5	3	3	2.5	1.5
77	0.175		4.5	2	1.5	1	3.5	1
65	0.172		4.5	5	4	3.5	3.5	3.5
76	0.162		10		6.5	3.5	4.5	9.5
78	0.151		7.5		4	2.5	3.5	12
85	0.148		4		2.5	1.5	2	2.5
52	0.141			3.5	2.5	3.5	3.5	3
68	0.139		8	1.5	5	5	3.5	2.5

82	0.139		6.5		3.5	2	3.5	
54	0.134		1.5	6.5	3	1.5	4.5	1
80	0.129				4.5	3	6.5	5.5
86	0.127				5.5	5	6	
79	0.126		9		3.5	4.5	4.5	5
69	0.114		8	6	4.5	3.5	3.5	3.5
83	0.113		10	2.5	1.5	3	2.5	2
82	0.111				5.5	6	5.5	
76	0.104		9	3.5	3	5	2.5	4.5
76	0.088			5	4.5	4	3.5	3.5
84	0.088				2.5	4	4.5	1.5
83	0.085		10		3.5	3.3	3.5	8.3
75	0.081			2.5	5	6	3.5	3
82	0.079				4.5	5	2.5	12
54	0.079		10		7.5	5.5	5.5	3
81	0.077				5	4	3.5	
73	0.076		6	2.5	3.5	2.5	2	1.5
38	0.075		4	2	1.5	2.5	2	0.5
53	0.073		10	10	3	3.5	4.5	1.5

Supplementary Table S2 Model performance on Test Dataset based on eGFR at presentation

Performance on Test Dataset	eGFR < 30, N = 90	eGFR ≥ 30, N = 388	Overall
ESKD incidence	26 (28.9%)	26 (6.7%)	52 (10.9%)
Accuracy	0.678	0.905	0.862
Sensitivity	0.846	0.462	0.654
Specificity	0.609	0.936	0.887
Positive Predictive Value	0.468	0.343	0.415

Abbreviations: eGFR, estimated Glomerular Filtration Rate; ESKD, End Stage Kidney Disease
